# Barriers and facilitators to state public health agency climate and health action: a qualitative assessment

**DOI:** 10.1186/s12889-023-14996-2

**Published:** 2023-01-21

**Authors:** Cat Hartwell, Sam Lovell, Jeremy J. Hess, Kathleen Dolan, Jamie Vickery, Nicole A. Errett

**Affiliations:** 1 Department of Environmental and Occupational Health Sciences, School of Public Health, University of Washington, Seattle, USA; 2 Department of Global Health, School of Public Health, University of Washington, Seattle, USA; 3 Department of Emergency Medicine, School of Medicine, University of Washington, Seattle, USA; 4 Center for Health and the Global Environment (CHanGE), School of Public Health, University of Washington, Seattle, WA USA; 5 Association of State and Territorial Health Officials, Arlington, VA USA

**Keywords:** Climate change adaptation, Public health, Policy

## Abstract

**Background:**

As the health implications of climate change become more apparent, agencies and institutions across the United States are developing recommendations for state and territorial health agencies (S/THAs) to implement evidence-informed climate and health adaptation strategies. The CDC established the Building Resilience Against Climate Effects (BRACE) framework in 2010 to encourage local and state public health engagement in climate change adaptation. However, even after a decade of the BRACE initiative, the elements that affect the adoption and implementation of climate and health programming by S/THAs are not well understood.

**Methods:**

Using an implementation science framework, this study sought to further understand and define the barriers and facilitators that determine the breadth and success of climate change and health activities undertaken by state health agencies (SHAs). We conducted focus groups with representatives from SHAs with and without climate and health programs, and analyzed data using the framework method for qualitative research.

**Results:**

This study identified funding, state and agency-level prioritization, staff capability and capacity, and political will and polarization as factors that influence the readiness for implementation and implementation climate for climate and health activities.

**Conclusions:**

As the impacts of climate change intensify, S/THAs will need to expand resources and capacity, and seek advocacy and assistance from external organizations in order to support the level of engagement required to strengthen climate resilience. Findings from this study have implications for public health policy and highlight potential pathways to expand support for climate and health activities in S/THAs in the U.S.

**Supplementary Information:**

The online version contains supplementary material available at 10.1186/s12889-023-14996-2.

## Background

There is growing recognition of the significant health implications of climate change. State and territorial (S/T) health agencies (S/THAs) have important roles to play in fostering healthy communities in the context of a changing climate [1]. Their roles are wide ranging, and include leveraging their own authorities to take direct action and using their expertise and influence to inform policy change.

In the United States, states “are the principal government entity responsible for protecting the public’s health”[1]. State-level public health is derived from the 10th amendment to the US Constitution, which reserves powers not explicitly granted to the federal government to the states [1]. Accordingly, states, territories and freely associated states have health departments or agencies responsible for public health, which may be either independent agencies that report directly to a board of health or to the state’s governor or agencies that exist as components of larger “superagencies” [1]. While agency organizational structures vary, each generally implements a package of programs and capabilities to meet the health needs of the communities they serve across the areas of communicable diseases control, chronic disease and injury prevention, environmental health, linkage with and access to clinical care, and maternal, child and family health services, and access to and linkage with clinical services [2]. As climate change causes more frequent and intense climate disasters, health systems and other public infrastructure will be disrupted, requiring S/THAs to adapt their approach to public health service delivery to meet the changing needs of communities [3].

To support S/THA engagement in climate and health adaptation, the U.S. Centers for Disease Control and Prevention (CDC) developed the five step Building Resilience Against Climate Effects (BRACE) framework: 1) Anticipate climate impacts and assess vulnerabilities; 2) Project the disease burden; 3) Assess public health interventions; 4) Develop and implement a climate and health adaptation plan; and 5) Evaluate impact and improve quality of activities [4]. The BRACE framework provides a structured approach for S/THAs and local health departments as they create, implement and assess health adaptation plans and identify gaps in vital public health services [5]. Preliminary reviews have found that this framework has strengthened collaboration activities and capacity building and has enabled better integration of health into climate planning  [6–8].

Through cooperative agreements, CDC’s Climate Ready States and Cities Initiative (CRSCI) has provided financial support to 18 and 11 state and local health agencies in 2010 and 2021, respectively, to implement BRACE, including a total of 17 SHAs [9]. While a recent survey of S/THAs showed the majority of those with climate and health programs had received federal support, the BRACE approach has been adapted or applied by jurisdictions that have not received CRSCI funding, demonstrating potential knock on effects. For example, the Swinomish Indian Tribal Community created an “indigenized BRACE,” or “i-BRACE”, which integrated the tribe’s health values [10]. Moreover, the Association of State and Territorial Health Officials has provided technical assistance and other support for U.S. territories and freely associated states to integrate BRACE strategies.

Regardless of BRACE funding, most U.S. S/THAs have begun to take action to address climate-related health impacts [5]. However, there is significant variation in readiness across states. A 2020 report found that while all 50 states and the District of Columbia have begun identifying climate-related exposures, fewer (44) have determined climate sensitive health outcomes or the causal pathways for climate-related hazards in their state (33), and even fewer (29) have established evidence-based interventions to safeguard against climate change related public health impacts [6]. In fact, many S/THAs climate activities have not progressed beyond the first step of identifying and assessing climate threats and vulnerabilities [11].

This variation in activity and readiness may be explained by systems-level and context-specific barriers that affect the extent of and approach to climate and health programming implemented by S/THAs [9]. In the context of climate change, inadequate resources and capacity are consistently cited as key obstacles to S/THA climate change programming by agency staff, but studies have also found other issues including: lack of clarity regarding the role of agencies in climate activities, uncertainty in projection of impacts, lack of evidence regarding climate and health interventions, political controversy, and the complexity of climate change [5, 10, 11]. Yet, there remains little evidence on how contextual factors influence the implementation of climate resilience-building strategies among S/THAs specifically [9].

Implementation science is a field of study examining the methods used to promote the uptake of evidence-based practices and research findings into practice to advance the effectiveness and quality of health services [12], and has been identified as a tool to understand and improve uptake of evidence-informed approaches to addressing climate and health [9]. Implementation science is grounded in several implementation frameworks, including those focused on implementation determinants, strategies, and outcomes, that seek to systematically assess implementation. This study used the popular Consolidated Framework for Implementation Research (CFIR), first published in 2009 and updated in 2022 (after our data collection and analysis was completed) to reflect user feedback, to guide the systematic assessment of multi-level determinants of implementation of climate and health programming (i.e., the innovation) [12, 13]. The CFIR provides a menu of constructs across five domains: the innovation (characteristics of the innovation being implemented); inner setting (the setting in which the innovation is being implemented); outer setting (the setting in which the inner setting exists); individuals (the roles and characteristics of individuals involved with implementation); and the implementation process [12, 13].

Accordingly, our study uses the CFIR to further understand and characterize the contextual barriers and facilitators influencing the extent of and approach to S/THA climate and health work. Findings from this research can inform national efforts to support and advance S/THA climate action, as well as actions undertaken by states and localities.

## Methods

The goal of this research is to identify climate and health activities being implemented by S/THAs across the United States, and to understand barriers and facilitators that promote or prevent the undertaking of climate and health activities. The University of Washington’s Human Subjects Division determined this research was human subjects research that qualifies for exempt status (Category 2) on September 27, 2021 (STUDY00014050).

### Data collection

The research team applied the CFIR, an implementation science framework, to inform the data collection and analysis process, including the development of a focus group facilitation guide and codebook to guide analysis.

We employed a purposive sampling strategy to recruit study participants with the help of partners from the Association of State and Territorial Health Officials (ASTHO). Potential focus group participants were identified via responses to a 2021 online survey on climate and health activities in 59 U.S. S/THAs [5].

Focus groups were conducted online using Zoom, with a duration ranging from approximately 50–60 min. In advance of the focus group discussions, potential study participants were provided background information on the goals of the study, read an informed consent statement, and given the opportunity to ask questions. The focus group facilitator obtained verbal consent from each participant before starting the discussion, including permission to record the conversation for note taking and transcription purposes. The focus group guide (Supplemental Materials) sought to broadly explore determinants across each of the CFIR five domains [12, 13].

### Data analysis

Recordings were transcribed verbatim by researchers (SL and CH). SL and CH then reviewed the notes and recordings individually to familiarize themselves with the data and to identify emergent themes. An initial codebook was developed deductively based on the focus group facilitator guide, and inductively based on specific CFIR constructs that were identified in the data through the data familiarization process [13]. Additional inductive codes, based on emergent themes identified through the data familiarization process that were not otherwise represented, were added to the codebook. Focus group transcripts were imported into NVivo, and codes were applied by researchers SL and CH. All coding decisions were made in tandem using a consensus-based approach.

The framework method for qualitative research, an analytical method that uses a matrix format consisting of codes (columns), cases (rows) and summarized data (cells) for organizing data so that themes can be compared and contrasted across units of analysis (here, focus group discussions), was used for analysis [14]. Researchers SL and CH each independently entered data extracted from the notes into the matrix and synthesized this data into an analytic memo where data was summarized across focus groups by code, highlighting commonalities and counterpoints [15]. Throughout each stage of analysis, researchers audited each other’s work.

## Results

Three focus groups were conducted between December 2021 and February 2022, including two with state health agencies (SHAs) that have climate and health programs (CHP) and one focus group with SHAs that do not have CHPs. There were 16 total SHA staff between the three focus groups. There were 6 participants in the first focus group of SHAs with CHPs, 4 participants in the second focus group with SHAs with CHPs, and 6 participants in the third focus group with SHAs without CHPs (including two from the same state). In total 15 unique states were represented across all U.S. Health and Human Services administrative regions except for regions 2 and 3, including 9 who had been recipients of CRSCI funding [9, 14]. No health agencies from U.S. territories or freely associated states participated in focus group discussions.

### Ongoing climate and health activities

Participants with CHPs described that the majority of their climate and health work has focused on building the evidence base around climate hazards through research and pilot programs; developing tools and resources, such as climate dashboards; internal and external collaborations to advance climate work; and assisting in climate-related response work, such as extreme weather events. Moving forward, these participants described wanting to focus work around equity, environmental justice and adaptation efforts. Participants without CHPs reported focusing their climate and health efforts on supporting groups that have funding to do climate work, participating on climate-related internal and external task forces, general environmental health-related activities, and preparation and response for extreme weather events.

#### Structural characteristics

Participants reported that their agencies have a range of full time staff for climate-related work (from 0 to 9.25) depending on funding, agency restrictions on the number of full-time employees that can be hired, and whether or not there is a dedicated CHP. Focus group participants with CHPs broadly reported more designated CHP staffing support than participants without a CHP. Many existing CHPs have operated for approximately ten years, when the BRACE program started. CHPs or climate-related activities are often housed in the same divisions as environmental health work.

#### Networks and communications

Participants in each focus group discussed coordination on climate-related issues within the SHA, across different agencies or programs (such as emergency preparedness, infectious disease), and through state-wide councils, committees, or groups. Regardless of a dedicated CHP, most participants described environmental health tracking activities and collaboration with environmental health staff as climate-related activities.

#### Engagement

Nearly all participants discussed engaging with academic centers on climate-related work, including information-sharing, data analysis and visualization, other research efforts, and in serving as trusted partners to communities. States with CHPs also described engagement with Governors’ offices to collaborate on climate-related initiatives and leadership.

### Determinants of climate and health program implementation

Participants identified funding, state and agency-level prioritization of climate and health, political will and polarization, and staff capability and capacity as factors that influence climate and health program implementation. These factors are most closely aligned with the following four CFIR constructs: readiness for implementation, implementation climate, external policies and incentives, and knowledge and beliefs surrounding climate and health activities (Table 1).

**Table 1:** Key climate and health implementation barriers and facilitators by CFIR construct

	Implementation Readiness	Implementation climate	External policies and incentives	Knowledge and beliefs
Funding	✓		✓	
State- and agency-level prioritization of climate and health	✓	✓	✓	✓
Political will and polarization	✓	✓	✓	
Staff capability and capacity	✓	✓		✓

#### Theme 1—Funding

Funding was discussed as a leading determinant of readiness for implementation, as it ensures agencies can hire and retain staff to carry out work within the agency, and have resources to develop and implement projects and to provide funding to community based organizations (CBOs), local health departments, and other partners. Nearly every CHP expressed the importance of funding: “it seems a bit flip to say more funding is what we need, but it really is what we need,” and “we would be very successful if we had additional funding, which we don’t.” States without CHPs also described how funding and the challenges of securing grants limited their ability to hire personnel, leaving them with minimal staff to handle all environmental health-related issues. States with CHPs noted that their collaboration with CBOs, communities, and local health jurisdictions, while ongoing, was challenged by lack of resources to provide to communities*.*

Federal funding, in particular, was noted by SHAs with CHPs as the primary determinant of CHP development and maintenance, with one SHA stating, “the climate and health program for [the state] has been very much tied to our BRACE funding. The program's existence and definition is related to that funding stream but also to political winds and our ability to be fully engaged on this topic.” However, one SHA with a CHP described how the structure of federal funding cycles does not fully match the longer term adaptation measures necessary for climate change, and suggested shifting away from shorter term funding cycles that promote shorter term projects.

When asked about what they might do with additional funding, SHAs discussed expanding staff capacity and subject matter expertise, developing grant programs for state agency collaborations, supporting local CBOs and agencies, investing in infrastructure for pilot projects and preparedness and programs, training and mentoring public health graduates, building a climate and health website, and focusing on climate justice-related programming.

#### Theme 2—State and agency-level prioritization of climate and health

The implementation climate within the state or the SHA itself, including the ability of staff to engage within and across state agencies and the relative prioritization of climate issues compared to other topics, can facilitate or hinder climate-related activities. Several SHAs with CHPs described the lack of representation of health officials in interagency and statewide climate task forces and officials not being invited to the table in such discussions. One shared that their request to join a Governor’s climate task force was denied and another stated, “we need more support from within our agency and more awareness and more knowledge, and more representation at the state agency convenings around climate change.” This extends to inclusion on issues with state legislatures: one SHA with a CHP described the importance of “getting ourselves at the table early,” to ensure they are able to provide input and shape climate and health-related bills and other legislative efforts. Another SHA with a CHP reported that climate and health related funding for their agency was left out of the state budget because of lack of representation during state legislative sessions. Several SHAs with CHPs also described issues with the current structure of climate-related work at the state level, and proposed reorganizing it in a way that normalizes public health involvement.

SHAs with and without CHPs expressed the need for external advocacy efforts to raise awareness about the importance of climate and health work and to provide resources. One SHA with a CHP stated, “Advocacy at the national level to draw more attention to the role of public health in climate change work and what we can bring to the table would be really helpful.” Such support from external advocates could also include providing technical resources, such as a national database for resource sharing, and communications materials, such as fact sheets for SHAs to use and adapt.

Consistently, SHAs with and without CHPs described how climate activities have been deprioritized internally as a result of the COVID-19 response and other competing public health issues: “The challenge for us has been that in a public health landscape that is very crowded with a lot of very hard issues, COVID of course, lead, opioids, a lot of other issues, it’s sort of getting a foothold and attention.” This de-prioritization was especially pronounced in SHAs without CHPs, which struggled to meet current baseline demands on their agencies, let alone engage in “extra” work related to climate. This was described as particularly challenging for SHAs in which environmental health staff had other time-intensive work on urgent issues such as Superfund sites.

Leadership support and engagement, or lack thereof, was also described to impact an agency’s readiness, with one SHA with a CHP describing, “we’ve had more or less supportive senior management and administrations for the work. Our ability to do the work and talk about the work is certainly tied to that, as it is for lots of things.” Particularly important is leadership engagement and support for climate work from governors, with one SHA with a CHP noting “we are really fortunate to have a pro-climate governor and that kind of trickles down through leadership whether people want it to or not.” Conversely, two SHAs without CHPs explained that resistance from their leadership actively prevented them from even seeking funding for climate-related activities. When asked about what they might do with additional funding, SHAs discussed increasing staff support, subject matter expertise, developing grant programs for state agency collaborations, and other activities they could engage in to broaden capacity and reach.

#### Theme 3—Political will and polarization

SHAs described political will as extremely influential in climate and health programming regardless of CHPs. However, polarization around climate change limiting programming was more pronounced among SHAs without CHPs, with one noting, “politics is probably our biggest issue. It’s hard to even apply for grants that have the phrase climate change in there. And if you want to work on climate change and you don’t have funding, that’s extra tricky to sell. So that has been a barrier.” This quote provides an example of how the political climate in a state may preclude climate and health activities or the development of a CHP, in this instance by way of restricting funding pursuits. Other participants explained that state-level political leadership can either help, for example through supportive executive orders, or hinder, for example through exclusion from state budgets, climate and health programming.

Further, changes in state-level political leadership and administrations can either stall or accelerate climate activities in SHAs with CHPs. As one participant shared, “Under our new governor, we’ve really had a chance to expand and work with other agencies and really raise the awareness of climate and health, that there is a climate and health program in the Department of Health, and we’ve been working for quite a while.”

#### Theme 4—Staff capability and capacity

The attitudes of SHA staff towards the importance of climate and health activities, and their overall understanding of the connection between climate and health, impacts the implementation of climate related activities. Perceived self-efficacy, or believing that one has the ability or know-how to implement climate and health activities, was described as a challenge by some SHAs. In regards to climate health policy, one SHA with a CHP said, “I want to be sure I’m doing it well, and [developing climate policy] is not something I’ve had a lot of experience doing, even though it’s something we’ve been building for a long time.” Beyond implementation of activities, other SHAs expressed feeling a limited ability to communicate about climate related work as a state agency.

When asked what kind of support is needed to achieve the goals of the CHP, increasing staff capacity in key expertise areas such as epidemiology, GIS, and community engagement were noted as important needs. Additionally, SHAs expressed lacking support with technical assistance and information resources for communication purposes. One SHA without a CHP said, “we could use some, maybe, direction or ideas as far as expanding programs … or sharing of data and sharing of ideas. Everything that we’ve done, we’ve done for years, and we’ve done it the same way.” SHAs with and without CHPs expressed interest in receiving additional support including from groups like ASTHO.

Several SHAs both with and without CHPs suggested that academic institutions could lend support to these challenges by acting as a trusted voice and partner in community engagement, raising public awareness, helping to reduce polarization on issues, and advocating for climate and health. On this topic, one SHA without a CHP claimed, “the universities [could] be the mouthpiece and the spokesperson so that they’re the ones to get the praise or the ridicule and the state worker who’s doing the work is just hidden in the background but the work still gets done.” This participant describes a model in which academic institutions can fill a publicly facing role, sharing the latest research and communications on climate and health while simultaneously providing political protection to SHA staff. SHAs suggested that academic institutions could help overcome challenges attributed to limited capacity by assisting with the analysis, tracking, management and dissemination of data. SHAs also said that academic institutions can lend expertise and conduct training with state SHA staff on environmental health issues, therefore enhancing the knowledgebase and increasing a sense of self-efficacy within the agency.

Alternatively, some SHAs with CHPs emphasized the vast amount of work they have done in the climate and health space, even with limited resources in some cases, and stressed the importance of SHAs celebrating the successes of their climate and health activities to boost morale. One SHA with a CHP said, “I do think it’s almost disorienting to think about what we’ve done in the last ten years, and I would say… that [our state], is…at the forefront of what it is to do health and climate change adaptation work.”

## Discussion

As recognition of the impacts of climate change on health grows, the role of public health in establishing adaptive policies and programs becomes even more critical. Our study identified the ways in which SHA implementation of climate and health programming is affected by funding, state and agency-level prioritization of the issue, political will and polarization, and staff capability and capacity. As SHAs seek to build up and strengthen climate and health activities in response to growing threats, they will need additional capacity, resources, advocacy and support from external organizations (Table 2).

**Table 2:** Challenges and proposed solutions for climate and health activity implementation identified by study participants

Implementation challenges	Proposed solutions
Funding	Support from external organizations on identifying viable and sustainable funding sources. Expanded federal programs to support state and local-level climate and health programs
State and agency-level prioritization of climate and health	Advocacy at the national level from organizations like the National Association of County and City Health Officials (NACCHO), ASTHO, etc. to raise the profile of climate as an urgent issue Normalize inclusion of public health in climate related work Engage leaders in conversations early Adopt a “climate in all policies” approach
Political will and polarization	Advocacy at the national level from organizations like NACCHO, ASTHO, etc
Staff capability and capacity	Consider agency restructuring to better accommodate and support activities Develop guides to identify engagement strategies and outline responsibilities of different agencies in climate activities Academic partnerships specifically tailored towards collection, analysis, dissemination of data, and training to enhance expertise

Funding was consistently recognized by SHAs to be the leading barrier and facilitator to climate and health activities. Funding shortfalls and other challenges have been previously described to have resulted in stalled climate action in the health sector–with steps taken to identify and quantify health impacts, but lack of development and implementation of mitigation or adaptation actions by the public health community [16, 17]. Our study participants echoed this impact, describing the deleterious impacts of being “late to the climate change table.” Moreover, as identified in one focus group with SHAs with CHPs, the current federal funding model, that leverages short-cycle, intermittently offered competitive grants, promotes short-term engagements and smaller projects, whereas adaptation measures required to address the long-term implications of climate change demand broader scopes and longer timescales. SHAs in our study also pointed out a cycle in which foundational funding is necessary to be able to secure more funding to sustain climate and health activities, with these funds ensuring the staff resources are available to identify and apply for grants in a complex federal landscape. Long-term, stable funding is thus urgently needed to facilitate SHAs’ ability to “catch up” and meet the increasingly urgent population health demands of the climate crisis. In the short-term amid absence of additional financial resources, tools or resources to adapt or implement BRACE are necessary, which has been described as being overly technical and “academic”[18].

At the global level, a major funding gap still exists to spur needed research and programs on climate change and health; adaptation funding for health systems constitutes less than 1% of global funding for climate change adaptation [16]. To the best of our knowledge, no such estimate of the parallel gap in the U.S. has been estimated. After taking office, President Biden identified climate change as a top priority and included specific climate and health funds in his administration’s initial budget request [18]. However, there has yet to be substantive increases in access to climate and health funds by S/THAs. There is an urgent need for the U.S. gap to be estimated in order to establish baseline data that can be used to urge state and federal policymakers to provide additional funding for adaptation.

Our research identified staff capability and capacity as another challenge for the implementation of climate and health activities for SHAs, in alignment with prior qualitative research among U.S. local health officials that identified competing local priorities and staff and funding constraints as the top impediments to their agency’s engagement in climate and health [18]. Lack of SHA climate and health expertise may be compounded by loss of institutional knowledge associated with large-scale anticipated retirements among the public health workforce, along with workforce burnout due to COVID-19 [11, 16, 17]. While SHAs reported maximizing the constrained resources they have, our findings affirm previous literature highlighting the limited capacity of SHAs in general as a deterrent to engagement in climate and health [10]. As one SHA with a CHP with one full-time employee suggested, “Imagine, how much more could be done if the necessary resources were provided.” Funding enables SHAs to hire and retain staff, thus preserving institutional knowledge, and funding can also support community partners doing frontline work. In the absence of additional resources, SHAs may consider mainstreaming climate and health work by identifying alignments with existing areas of work, for example public health emergency preparedness and response, echoing calls from a qualitative study employing interviews with state and local health officials in the U.S. Pacific Northwest [15]. In the medium to long term, public health training programs, including those offered in continuing education and university-based settings, can train the workforce needed for the future by integrating competencies related to climate change and health, across the broad domains of “climate and environment sciences, drivers of climate change, evidence, projections and assessments; iterative risk management; mitigation, adaptation and health co-benefits; and collective strategies” that leverage mutual agreements and frameworks across multiple levels of government [19].

Resources and infrastructure are also necessary to support networking among public health practitioners with differential levels of experience in climate and health programming. Indeed, qualitative research identified the most common area of capacity development for climate and health called for by local health officials was learning from their peers [18]. Furthermore, an evaluation of a program that provided 12 urban local health agencies with support for networking and relationship development, information sharing, communication, and learning, found that the efforts springboarded projects that enhanced their agencies’ climate change-related engagement, community partnerships, and integration into climate planning activities within their jurisdictions [20].

Our findings underscore that the relative priority of climate change and health among SHAs does not align with the magnitude of the existential threat, and that lack of political support may further disincentivize or inhibit climate and health program implementation [18]. Prior qualitative and survey research of state and local health officials have also consistently found that, while officials identify climate change as a serious problem, it is not among their top priorities [8, 15, 21]. Furthermore, a 2010 survey of Environmental Health Directors (EHDs) at local, state, and territorial public health agencies in the U.S. explored their environmental attitudes, including knowledge and beliefs surrounding climate change impacts and actions [22]. The researchers received 194 full survey responses from across the U.S. over a four-week period and found a relationship between the political views of EHDs and their environmental attitudes, pertinent because of the individual influence EHDs maintain over public health policy more broadly, and more specifically with the development and implementation of climate change adaptation programs and activities [22]. Our survey supports previous findings that political and public support for climate adaptation, or lack thereof, can make or break SHA engagement in climate and health activities. As one focus group participant without a CHP said, “unfortunately, public support may not always be there for us as well, which can cause some issues in reaching out for such issues as climate change and funding and support for that.”

The structure of state and federal governments, which are often fragmented and organized into specialized policy areas, has been shown to be inconducive to robust and effective climate adaptation strategy [23], and that more interagency and external collaboration may help SHAs incorporate climate adaptation into existing activities more effectively [15, 21, 24]. Accordingly, SHAs may consider developing resources to promote collaborations with external partners for climate and health work, including those based at academic institutions and professional associations.

### Limitations

Our study lacked representation from health agencies in the U.S. territories and freely associated states, had a relatively small sample size, and had greater representation of SHAs with versus without CHPs, which may impact the generalizability of the results. Additionally, as the unit of analysis in our study is the focus group, we are unable to compare and contrast perspectives of individual study participants. Future research on this topic should seek out the perspectives of territorial health agencies to understand the unique challenges they face with the implementation of climate change and health programming.

While our study was undergoing peer review, the CFIR, which was originally developed in the context of health services research, was updated to reflect user feedback from its application in diverse settings [12, 13]. The update includes refinements to existing constructs and addition of new constructs [12]. Future research should explore how these new constructs, particularly critical incidents (“Large-scale and/or unanticipated events disrupt implementation and/or delivery of the innovation”) and local conditions (“Economic, environmental, political, and/or technological conditions enable the Outer Setting to support implementation and/or delivery of the innovation”), influence implementation of state-level climate and health programming [12].

## Conclusions

The health impacts of climate change require urgent engagement of public health agencies across all levels of government. Through three focus groups with 16 public health officials representing 15 U.S. SHAs, we identify that SHA barriers to implementation of climate and health programming are focused within the domains of funding, state and agency-level prioritization of climate and health, political will and polarization, and staff capability and capacity. Stable and long-term funding, opportunities for peer learning and networking, and pathways and guidance to support interagency collaborations are necessary to build SHAs' capability and capacity to respond to the climate crisis with the urgency it demands. Fortifying relationships with national agencies and academic partners can augment SHA capabilities, support evaluation necessary to demonstrate return on investment of climate and health programming, and ensure a competent workforce with the knowledge, skills, and abilities to tackle the health challenges of the future .

## Supplementary Information

**Additional file 1.** APPENDIX - Focus Group Facilitator Guide.

## Data Availability

The datasets generated and/or analyzed during the current study are not publicly available due to concerns of privacy and confidentiality but are available from the corresponding author on reasonable request.

## Abbreviations

ASTHO: Association of State and Territorial Health Officials
BRACE: Building Resilience Against Climate Effects
CFIR: Consolidated Framework for Implementation Research
CHP: Climate and Health Program
S/T: State and Territorial
S/THA: State and Territorial Health Agency
SHA: State Health Agency

## References

[1] Institute of Medicine (US) Committee for the Study of the Future of Public Health. Summary of the Public Health System in the United States. Washington (DC): National Academies Press (US); 1988. Available from: ncbi.nlm.nih.gov/books/NBK218212/. Cited 2022 Dec 3.

[2] Revising the Foundational Public Health Services in 2022. Public Health National Center for Innovations. 2022. Available from: https://phnci.org/transformation/fphs. Cited 2022 Dec 3.

[3] Ebi KL, Vanos J, Baldwin JW, Bell JE, Hondula DM, Errett NA (2021). Extreme weather and climate change: population health and health system implications. Annu Rev Public Health..

[4] Marinucci GD, Luber G, Uejio CK, Saha S, Hess JJ (2014). Building resilience against climate effects—a novel framework to facilitate climate readiness in public health agencies. Int J Environ Res Public Health..

[5] CDC’s Climate-Ready States & Cities Initiative. Centers for Disease Control and Prevention (CDC). 2022. Available from: https://www.cdc.gov/climateandhealth/climate_ready.htm. Cited 2022 Dec 1.

[6] Eidson M, Clancy KA, Birkhead GS (2016). Public health climate change adaptation planning using stakeholder feedback. J Public Health Manag Pract..

[7] Sheehan MC, Fox MA, Kaye C, Resnick B (2017). Integrating health into local climate response: lessons from the U.S. CDC climate-ready states and cities initiative. Environ Health Perspect..

[8] Grossman E, Hathaway M, Bush KF, Cahillane M, English DQ, Holmes T (2019). Minigrants to local health departments: an opportunity to promote climate change preparedness. J Public Health Manag Pract..

[9] Climate-ready states & cities initiative grant recipients. Centers for disease control and prevention (CDC). 2022. Available from: https://www.cdc.gov/climateandhealth/crsci_grantees.htm.

[10] Schramm PJ, AlJanabi AL, Campbell LW, Donatuto JL, Gaughen SC. How indigenous communities are adapting to climate change: insights from the climate-ready tribes initiative. Health Aff. 2020;39(12):2153–9. Available from: https://www.ncbi.nlm.nih.gov/pubmed/33284701. 10.1377/hlthaff.2020.00997 PMC8931667 33284701

[11] TFAH. The Impact of Chronic Underfunding on America’s Public Health System: Trends, Risks, and Recommendations. Trust for America’s Health. 2020. Available from: https://www.tfah.org/report-details/publichealthfunding2020/. Cited 28 May 2020.

[12] Damschroder LJ, Reardon CM, Widerquist MAO, Lowery J (2022). The updated consolidated framework for implementation research based on user feedback. Implement Sci..

[13] Damschroder LJ, Aron DC, Keith RE, Kirsh SR, Alexander JA, Lowery JC (2009). Fostering implementation of health services research findings into practice: a consolidated framework for advancing implementation science. Implement Sci..

[14] HHS Regional Offices. U.S. Department of Health & Human Services. 2021. Available from: https://www.hhs.gov/about/agencies/iea/regional-offices/index.html. Cited 30 Nov 2022.

[15] Doubleday A, Errett NA, Ebi KL, Hess JJ (2020). Indicators to guide and monitor climate change adaptation in the US Pacific Northwest. Am J Public Health..

[16] Leider JP, Coronado F, Beck AJ, Harper E (2018). Reconciling supply and demand for state and local public health staff in an era of retiring baby boomers. Am J Prev Med..

[17] Maani N, Galea S (2020). COVID-19 and Underinvestment in the public health infrastructure of the United States. Milbank Q..

[18] Zuber A. The University of North Carolina at Chapel Hill. 2018. Available from: https://search.proquest.com/openview/65de608cb15bfc9021b221312704d868/1?pq-origsite=gscholar&cbl=18750 30689332

[19] Jagals P, Ebi K (2021). Core competencies for health workers to deal with climate and environmental change. Int J Environ Res Public Health..

[20] Rudolph L, Maizlish N, North S, Dervin K (2020). A public health learning collaborative on climate change for urban health departments, 2016–2018. Public Health Rep..

[21] Austin SE, Ford JD, Berrang-Ford L, Biesbroek R, Ross NA (2019). Enabling local public health adaptation to climate change. SocSci Med.

[22] Syal SS, Wilson RS, Crawford JM, Lutz J (2011). Climate change and human health—what influences the adoption of adaptation programming in the United States public health system?. Mitigation Adapt Strat Glob Change..

[23] Huang C, Vaneckova P, Wang X, Fitzgerald G, Guo Y, Tong S (2011). Constraints and barriers to public health adaptation to climate change: a review of the literature. Am J Prev Med..

[24] Boyer CJ, Bowen K, Murray V, Hadley J, Hilly JJ, Hess JJ (2020). Using implementation science for health adaptation: opportunities for Pacific Island countries. Health Aff..

