# Supplementary material for: Barriers and facilitators to state public health agency climate and health action: a qualitative assessment
Source: BMC Public Health. 2023 Jan 21;23:145. doi: 10.1186/s12889-023-14996-2 (PMC9859738; doi:10.1186/s12889-023-14996-2)
Supplement: Supplementary file 1 — Additional file 1. APPENDIX - Focus Group Facilitator Guide. [file 12889_2023_14996_MOESM1_ESM.docx]

**APPENDIX:
Focus Group Facilitator Guide**

Welcome

- Thank you for agreeing to participate in our focus group. My name is [name] and I am a [role] in the [organization] **.**  I am part of an Association of State and Territorial Health Officials (ASTHO) and University of Washington team working to understand how state health departments are currently addressing climate change.
- The purpose of this focus group is to help us understand the types of planning, activities, and partnerships that your health department is undertaking related to climate change, as well as any factors that influence the development, uptake, and sustainment of climate change activities.

Explanation of the process

- We are using a focus group format to help understand the context behind the survey answers you provided, and to explore topics in more detail. We are seeking to learn from you the positive and the negative factors influencing the development, uptake, and sustainment of climate change activities. There are no wrong answers. We’re not trying to achieve consensus; we’re gathering information.
- The focus group will last about one hour.
- We will be taking notes and recording the focus group so that we can refer to the discussion later.
- We may write up our findings in a report or for publication in a peer-reviewed journal. We will not refer to you by name or agency in any report or publication without your prior explicit permission.
- Your participation is voluntary. You can refuse to answer any question, and you can leave the focus group at any time. You will not be penalized for not answering any question or for leaving the focus group.

Ground Rules

- We do have a few ground rules. We hope everyone will participate and chime-in during the discussion. Information provided in the focus group must be kept confidential. Please do not share what was said or who was here. Please stay with the group and avoid distractions.

Questions and consent

- Does anyone have any questions before we begin?
- Do you consent to participate in our focus group? [Ask everyone to provide a verbal “yes.”]
- Turn on Tape Recorder

**Focus Group WITH Climate & Health Program**

1. Before we begin, I’d like to go around the Zoom room and let everyone introduce themselves. Can you please share your first name, your role, and your organization?

*Next we would like to learn more about your agency’s climate and health program or activities.*

*[Inner setting-related questions: what’s happening w/in organization that influences implementation]*

Everyone in this focus group indicated in the survey that their jurisdiction currently does have a climate and health program. We also understand that some are in the process of developing a climate and health program. By climate and health program, we mean any program that addresses associations between climate and climate change and health and has at least 1 staff person designated to spend part of their time on climate change programming.

Prompt: Can you describe where your agency is in the development or implementation process of the climate and health program?
What kinds of climate-related planning, activities, and partnerships are already happening in your agency?

1. What are internal barriers to carrying out your climate change and health program?
2. What factors outside of your organization impact your ability to create or implement climate and health program?
   1. Prompt: political considerations, leadership
3. *Have you or your agency set goals related to the implementation of the climate change and health program? What are they*?

*Wrap-Up Questions*

1. **What support does your agency need to achieve your goals for your climate and health program?**
   1. Prompt: science information needs, funding, human resources, etc.
   2. Follow up: How can academic partners help you meet those needs?
2. As you know, ASTHO provides a variety of services for its members. What types of ASTHO services and support would be most helpful for your agency’s climate and health programs?
   1. Prompt: convenings, technical assistance, development of learning/training opportunities, peer-to peer mentorship opportunities, and travel and registration support for training and conferences.
      1. **ASTHO has launched a peer-to-peer climate and health fellowship wherein a S/THA that does not receive CDC funds is paired with a BRACE-funded state to accomplish a climate and health capacity building activity of their choosing. No funding is provided. Would you be able to dedicate 1 hour per month to serve as fellowship mentor? (Please feel free to put your response in the chat)**
      2. *Do you need additional FTEs to accomplish your agency's climate program activities/goals?*
         1. *Follow up: If so, what level of staffing would be sufficient?*
3. ASTHO Funding Priorities: If your agency were to receive federal funds for climate and health activities, what would you prioritize?
   1. What would you do with the funds?
4. Those are all the specific questions we have for you today. Is there anything else you’d like to tell us about?

**Questions for Focus Group WITHOUT Climate & Health Programs**

1. Before we begin, I’d like to go around the Zoom room and let everyone introduce themselves. Can you please share your first name, your role, and your organization?

*Next we would like to learn more about your agency’s climate and health activities.*

*[Inner setting-related questions: what’s happening w/in organization that influences implementation]*

Everyone in this focus group indicated in the survey that their jurisdiction currently does not have a climate and health program. By climate and health program, we mean any program that addresses associations between climate, climate change and health, and has at least 1 staff person designated to spend part of their time on climate change programming.

1. Can you describe what kinds of climate-related planning, activities, and partnerships are already happening in your agency?
2. What are internal barriers to carrying out your climate and health activities?
3. What factors outside of your organization impact your ability to create or implement climate and health activities?
   1. Prompt: political considerations, leadership

*Wrap-Up Questions*

1. What support does your agency need to continue its climate and health activities?
   1. Prompt: science information needs, funding, human resources, etc.
   2. Follow up: How can academic partners help you meet those needs?
2. As you know, ASTHO provides a variety of services for its members. What types of ASTHO services and support would be most helpful for your agency’s climate and health activities?
   1. Prompt: convenings, technical assistance, development of learning/training opportunities, peer-to peer mentorship opportunities, and travel and registration support for training and conferences.
   2. **ASTHO has launched a peer-to-peer climate and health fellowship wherein a S/THA that does not receive CDC funds is paired with a BRACE-funded state to accomplish a climate and health capacity building activity of their choosing. No funding is provided. Would you be interested in participating in this fellowship?**
   3. **ASTHO has heard that S/THAs that do not currently receive CDC funding would like to apply for BRACE funding but encounter challenges such as insufficient time and staffing to complete the application, and unmet application requirements.**
      1. **Have you considered applying for BRACE but declined? If so, why?**
      2. **If there was an opportunity to apply for a starter BRACE grant program would you apply?**
   4. ***What level of staffing would be necessary to initiate a climate and health program in your agency?***
3. **If your agency were to receive federal funds for climate and health activities, what would you prioritize?**
   1. **What would you do with the funds?**
4. Those are all the specific questions we have for you today. Is there anything else you’d like to tell us about?

​​​
